# Effects of thyroxine and 1-methyl, 2-mercaptoimidazol on phosphoinositides synthesis in rat liver

**DOI:** 10.1186/1476-511X-3-28

**Published:** 2004-12-10

**Authors:** Nataliya A Babenko, Oksana A Krasilnikova

**Affiliations:** 1 Department of Physiology of Ontogenesis, Institute of Biology, Karazin Kharkov National University, 4, Svobody pl., Kharkov, 61077, Ukraine

## Abstract

**Background:**

Phosphoinositides mediate one of the intracellular signal transduction pathways and produce a class of second messengers that are involved in the action of hormones and neurotransmitters on target cells. Thyroid hormones are well known regulators of lipid metabolism and modulators of signal transduction in cells. However, little is known about phosphoinositides cycle regulation by thyroid hormones. The present paper deals with phosphoinositides synthesis de novo and acylation in liver at different thyroid status of rats.

**Results:**

The experiments were performed in either the rat liver or hepatocytes of 90- and 720-day-old rats. *Myo*-[^3^H]inositol, [^14^C]CH_3_COONa, [^14^C]oleic and [^3^H]arachidonic acids were used to investigate the phosphatidylinositol (PtdIns), phosphatidylinositol 4-phosphate and phosphatidylinositol 4,5-bisphosphate (PtdInsP_2_) synthesis. 1-methyl, 2-mercaptoimidazol-induced hypothyroidism was associated with the decrease of *myo*-[^3^H]inositol and [^3^H]arachidonic acids incorporation into liver phosphoinositides and total phospholipids, respectively. The thyroxine (L-T_4_) injection to hypothyroid animals increased the hormones contents in blood serum and PtdInsP_2 _synthesis de novo as well as [^3^H]arachidonic acids incorporation into the PtdIns and PtdInsP_2_. Under the hormone action, the [^14^C]oleic acid incorporation into PtdIns reduced in the liver of hypothyroid animals. A single injection of L-T_4 _to the euthyroid [^14^C]CH_3_COONa-pre-treated animals or addition of the hormone to a culture medium of hepatocytes was accompanied by the rapid prominent increase in the levels of the newly synthesized PtdIns and PtdInsP_2 _and in the mass of phosphatidic acid in the liver or the cells.

**Conclusions:**

The data obtained have demonstrated that thyroid hormones are of vital importance in the regulation of arachidonate-containing phosphoinositides metabolism in the liver. The drug-induced malfunction of thyroid gland noticeably changed the phosphoinositides synthesis de novo. The L-T_4 _injection to the animals was followed by the time-dependent increase of polyphosphoinositide synthesis in the liver. The both long-term and short-term hormone effects on the newly synthesized PtdInsP_2 _have been determined.

## Background

The phosphoinositides is a family of lipids which members play an essential role in the receptor-mediated intracellular signaling cascades, vesicle trafficking and cytoskeletal rearrangements [for review see [1,2]] and, therefore, are crucial for the adaptation and survival of cells. Because of their importance in numerous signaling events, the phosphoinositides require an absolutely tight temporal and spatial regulation of synthesis and degradation, enabling the cell to maintain organelle identity and housekeeping functions. A rapid agonist-dependent burst of phosphoinositides biosynthesis was the first feature of the polyphosphoinositide signaling pathway to be discovered, illustrating that synthesis is tightly coupled to degradation.

The two pools of phosphoinositides are supposed to exist in the cells [3,4]. One of these is sensitive to the hormone-induced hydrolysis, and the other is hydrolysis-insensitive. The biosynthesis of phosphatidylinositol (PtdIns) is the two-component process composed of cytidyltransferase followed by a synthase [for review see [5,6]]. The first detailed study of the occurrence and localization of the PtdIns synthase activity in various tissues from guinea pig was carried out by Benjamins and Agranoff [7]. They detected the enzyme activity in all the tissues tested, including brain, liver, kidney, heart, lung and spleen. The PtdIns synthase activity was also detected in the endoplasmic reticulum, plasma membranes, Golgi apparatus and nuclei. The hormone-sensitive pool of phosphatidylinositol 4,5-bisphosphate (PtdInsP_2_) in the plasma [8] and nuclear [9] membranes re-synthesized in these membrane fractions. The PtdIns synthesized de novo in the endoplasmic reticulum could be converted to the glycosylPtdIns or transferred by transfer proteins to other cellular compartments where the lipid was used for the phosphatidylinositol 4-phosphate (PtdInsP), PtdInsP_2 _and other polyphosphoinositides production [10,11].

The regulation of the key enzymes of the PtdIns synthesis pathway: the CDP-diacylglycerol (DAG) synthase and PtdIns synthase were examined [12]. Expression of the PtdIns synthase gene caused the overproduction of the both of the PtdIns synthase and PtdIns:inositol exchange reactions, indicating that the gene encode the both enzymes. However, the overexpression of PtdIns synthase or CDP-DAG synthase alone or in combination in the COS-7 cells did not enhance the rate of the PtdIns biosynthesis and did not result in a significant proportional increase in the CDP-DAG and PtdIns cellular levels. The CDP-DAG synthase activity was inhibited by polyphosphoinositides in vitro [13]. This makes possible to suggest that these end products of the pathway may function as the feedback inhibitors of PtdIns biosynthesis in vivo. The PtdIns synthase activity has been shown to be upregulated after the hormone-induced phospholipase C (PLC) mediated hydrolysis of phosphatidylinositol-polyphosphates [14,15]. The efficiency of the PtdIns synthesis is dependent on the CDP-DAG fatty acids composition [for review see [5,6]]. In the bovine brain, the CDP-DAG has been shown to present predominantly as the 1-steroyl, 2-arachidonyl which are also the main PtdIns components.

Thyroid hormones are of vital importance in maintaining the initial level of phospholipids in cell membranes and fatty acids composition of the lipids. On the other hand, there is a little literature regarding the hormone regulation of phosphoinositides exchange. The levels of [^32^P]phosphoinositides and inositol 1,4,5-trisphosphate were found to be significantly lower in the hypothyroid rat hearts [16]. The effect of hypothyroidism on the insulin- and epinephrine-stimulated phosphoinositide metabolism has been investigated in the rat adipocytes [17]. The hypothyroidism enhanced the insuline-mediated phosphoinositides synthesis. The hypothyroidism caused a significant increase in both the basal and ouabain-stimulated accumulation of [^3^H]inositol phosphate in the hypothalamic slices, whereas the thyroxine (L-T_4_) completely restored the hypothalamic [^3^H]inositol phosphate formation [18]. The results indicate that the negative feedback action of the thyroid hormone may occur at a post-receptor site in the hypothalamus. The thyroid hormones might participate in regulating the muscarinic cholinergic neurotransmission in the adult rats striatum via the stimulatory action on the inositol phosphate formation in the [^3^H]inositol pre-labeled tissue [19]. Thus, it becomes evident that the thyroid gland malfunction leads to the prominent disturbances of signal transduction in the adipocytes and nervous cells via the changes of phosphoinositides metabolism.

In the paper, we examined the influence of the drug- and L-T_4_-altered thyroid status of animals on the phosphoinositides synthesis in the liver. Thus, it was determined that the hypothyroidism decreased considerably the levels of the newly synthesized lipids in the liver but thyroid hormones increased the synthesis of the polyphosphoinositides de novo and the arachidonic acid incorporation into the PtdIns and PtdInsP_2_. A single injection of the L-T_4 _to the euthyroid rats lead to the rapid and transient decrease of the newly synthesized PtdInsP_2 _followed by the increase of the PtdIns and PtdInsP_2 _levels in the liver.

## Results and Discussion

The present paper considers the influence of thyroid functional status on the phosphoinositides synthesis in the liver. To determine the role of thyroid hormones in the regulation of phosphoinositides synthesis de novo and lipids fatty acid remodeling, the euthyroid, MMI (1-methyl, 2-mercaptoimidazol)- and MMI+L-T_4_-treated rats and intact animals after the single hormone injection has been studied.

It has been reported that the L-T_4 _induced hydrolysis of PtdInsP_2 _and inositol phosphates and DAG formation at the early stages of hormone action on the [^14^C]oleic and [^14^C]linoleic acid pre-labeled hepatocytes of adult 90-day-old rats [20,21]. The L-T_4_-mediated PLC activation was accompanied by the protein kinase C (PKC) translocation to membranes [20] and PKC dependent stimulation of mitogen-activated protein kinase [22] and acylation of phospholipids and triacylglycerol synthesis [23]. The PtdInsP_2_-specific PLC activation in the L-T_4_-stimulated cells was a short-lived event. The hormone-stimulated rise in inositol 1,4,5-trisphosphate (Ins(1,4,5)P_3_) was followed by its conversion into the biologically inactive inositol 1,4 -bisphosphate and inositol 1-phosphate [21].

**Figure 1:**
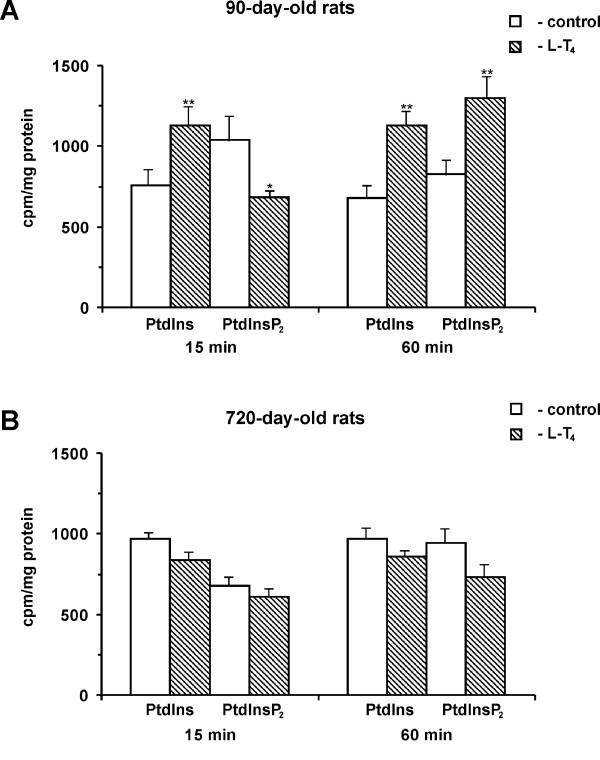
Short-term effects of L-thyroxine on the newly synthesized phosphoinositides levels in liver. Panel A – 90-day-old rats; panel B – 720-day-old rats. The liver lipids were labeled by [^14^C]CH_3_COONa as described in "Materials and Methods". The L-T_4 _(200 μg/100 g weight) was injected to the normal rats 15 and 60 min prior to killing. The lipids were extracted and separated as described in "Materials and Methods" and the radioactivity was determined by a liquid scintillation counter. Results are mean ± S.E. of six experiments performed in duplicate.

It is known that after the receptor-triggered hydrolysis of phosphatidylinositol-polyphosphates the phospholipid must be resynthesized in order to maintain a constant level of phosphoinositides in the membranes. A single injection of L-T_4 _to the 90-day-old rats leads to the rapid and sustained increase in the L-T_4 _and triiodothyronine (L-T_3_) levels in blood serum (Table 1), the content of the newly synthesized PtdIns and transient decrease the PtdInsP_2 _level in the [^14^C]CH_3_COONa-pre-labeled liver, which was followed by the polyphosphoinositide level increase (Figure 1A). Taking into account that the PtdInsP_2 _suppresses the key enzymes of PtdIns synthesis [13], the transient hormone-stimulated and phospholipase C-mediated [20,21] drop of the newly synthesized polyphosphoinositide in the liver cells was supposed to be the stimulus for the PtdIns synthesis. The L-T_4 _administration to the old 720-day-old rats did not change the newly synthesized PtdIns and PtdInsP_2 _levels in the liver (Figure 1B) although increased thyroid hormones contents in blood serum (Table 1). It has been shown that the thyroid hormones are unable to stimulate rapidly the phospholipase C in the [^14^C]CH_3_COONa-pre-labeled liver slices and hepatocytes of old animals [24]. These observations, together with the earlier data [20,21], suggest that the L-T_4 _stimulates rapidly and nongenomicaly the phosphoinositides degradation and resynthesis in the liver of adult rats and does not act on the phosphoinositides synthesis in the cells with the disability of polyphosphoinositide signaling at old age.

**Table 1:** Thyroxine and triiodothyronine contents in the blood serum of the rats of different thyroid states and age.

Animals		T_4_	T_3_
Adult control		128 ± 3.80	1.21 ± 0.20
Adult MMl-treated		29.5 ± 5.00**	0.83 ± 0.01*
Adult MMl+T4-treated		57.3 ± 7.30***	1.61 ± 0.10***
Adult T4-treated	1	654 ± 50.0**	5.78 ± 0.20**
	2	675 ± 98.5**	15.9 ± 4.10**
Old control		70.7 ± 11.6	1.37 ± 0.07
Old T4-treated	1	730 ± 15**	5.8 ± 1.5**
	2	537 ± 128**	4.9 ± 0.0**

**Figure 2:**
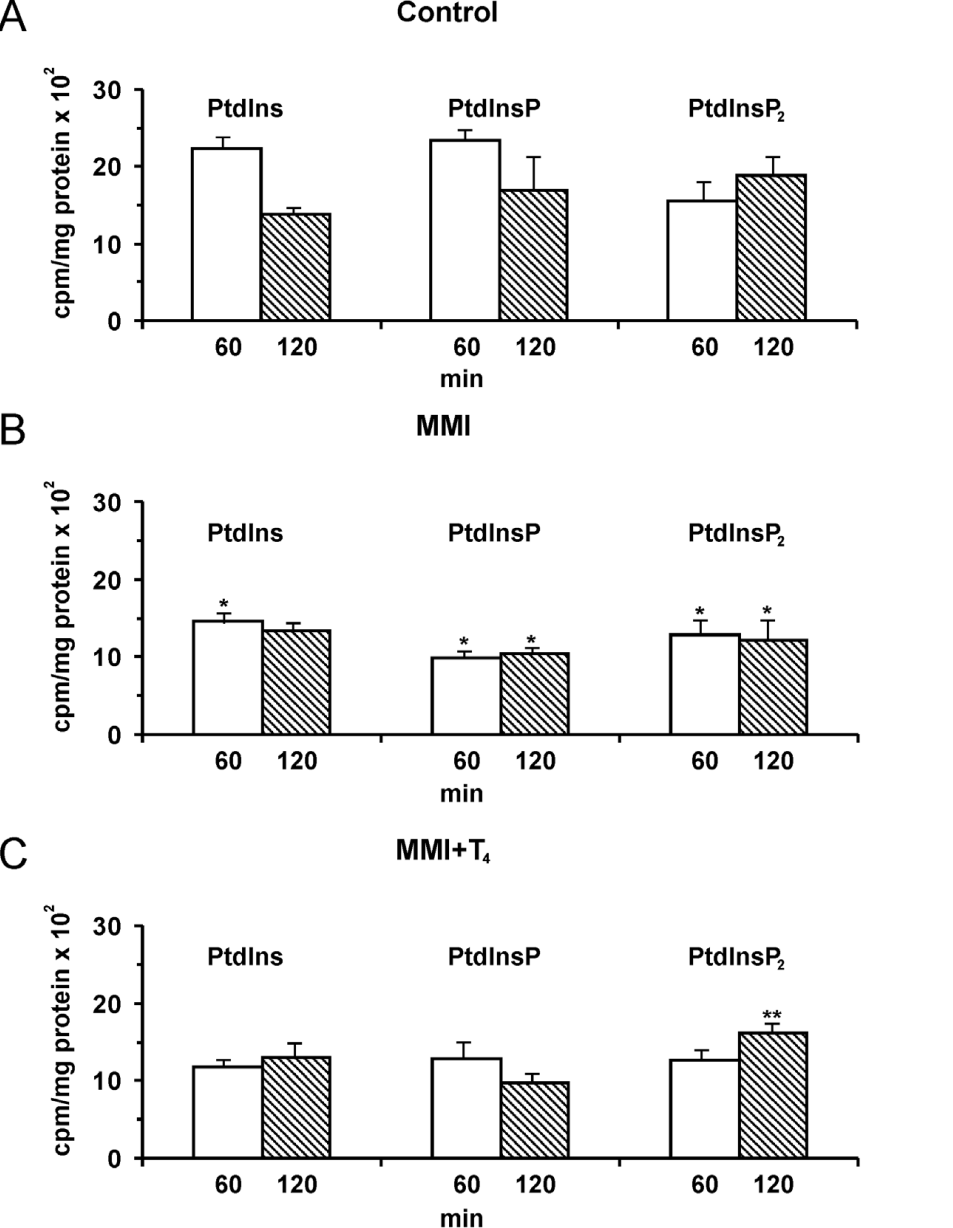
Phosphoinositides synthesis in the liver of rats of different thyroid status. Panel A shows *myo*-[^3^H]inositol incorporation into the lipids of control rats. Panel B shows the lipid precursor incorporation into the lipids of liver of MMI-treated animals. Panel C shows effect of L-T_4 _on phosphoinositides synthesis in liver of MMI-treated rats. Treatment of the 90-day-old rats by MMI was performed as described in ''Materials and Methods''. L-T_4 _(200 μg/100 g weight) was injected to the MMI-treated rats 48 h prior to killing. Control rats received the same volume of 0.9% NaCl. Liver slices were incubated in the presence of the *myo*-[^3^H]inositol and lipids were extracted and separated as described in ''Materials and Methods'' and the radioactivity was determined by liquid scintillation counter. Results are mean ± S.E. of 6 – 8 individual experiments performed in duplicate. *P < 0.05 vs. control, **P < 0.05 vs. MMI-treated rats.

**Figure 3:**
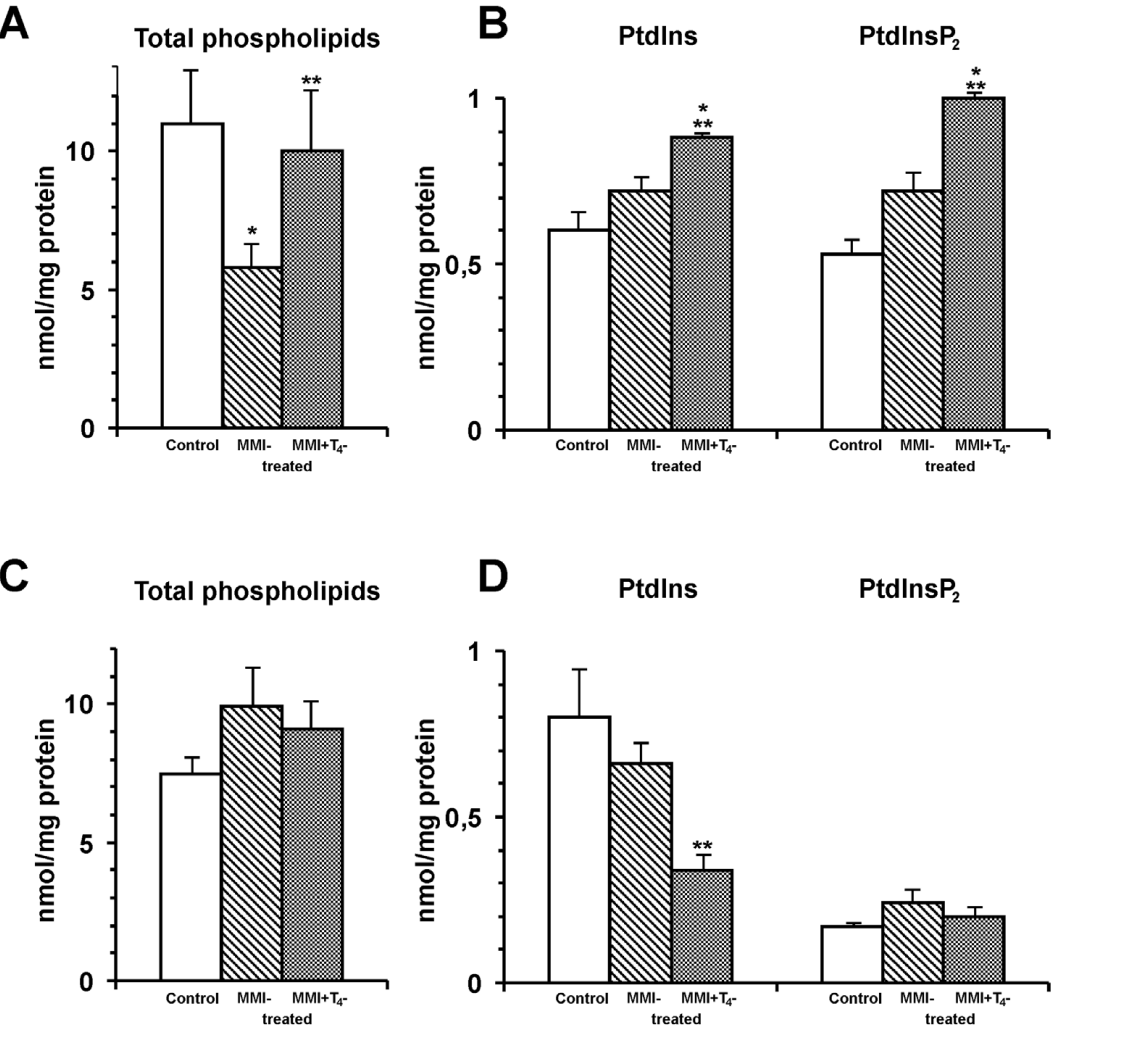
Incorporation of [^3^H]arachidonic and [^14^C]oleic acid into liver total phospholipids and phosphoinositides of rats of different thyroid status. Panel A and B show [^3^H]arachidonic acid incorporation into total phospholipids and phosphoinositides of rats of different thyroid status, respectively. Panel C and D show the [^14^C]oleic acid incorporation into total phospholipids and phosphoinositides of rats different thyroid status, respectively. Treatment of the 90-day-old rats by MMI was performed as described in "Materials and Methods". L-T_4 _(200 μg/100 g weight) was injected to the MMI-treated rats 48 h prior to killing. Control rats received the same volume of 0.9% NaCl. Liver slices were incubated in the presence of the [^3^H]arachidonic or [^14^C]oleic acid and lipids were extracted and separated as described in ''Materials and Methods'' and the radioactivity was determined by liquid scintillation counter. Results are mean ± S.E. of 6 – 8 individual experiments performed in duplicate. *P < 0.05 vs. control, **P < 0.05 vs. MMI-treated rats.

The T_3 _and T_4 _contents in the blood serum were determined by radioimmunoassays kits. The amount of thyroid hormones in serum was represented as nmol per liter. Treatment of the rats by MMl was performed as described in "Materials and Methods". T_4 _(200 mg/100 g weight) was injected to the MMl-treated rats 48 h prior to killing or normal rats 15 (1) and 60 (2) min prior to killing. Results are mean ± S.E. of 6–8 individual experiments performing in duplicate. In the Tables 1–2 and Figures 1–3 one experiment is equivalent to measurement of the parameters studied in a sample of liver of single animal. * P < 0.05 vs. control, ** P < 0.001 vs. control, *** P < 0.05 vs. MMl-treated rats.

In addition to its established role as a precursor for the signaling molecules (Ins(1,4,5)P_3 _and inositol 3,4,5-trisphosphate), the PtdInsP_2 _is now recognized as an important plasma membrane signal that activates the PLD [for review see [25]] and thus establishes the sites for vesicular trafficking, membrane movement and cytoskeletal assembly. It has been demonstrated in the mammalian cells that the PtdInsP_2 _is a membrane-associated cofactor of PLD [26,27]. The rapid stimulation of the PtdInsP_2_-specific PLC by the L-T_4 _in the rat hepatocytes of adult 90-day-old rats was followed by the prominent PLD activation [20]. The PLD response in the stimulated cells was reduced by the both PKC inhibitor and a high affinity ligand of PtdInsP_2 _– neomycin. Neomycin does not directly interact with PLC and PLD but interacts with the endogenous membrane PtdInsP_2_. Inclusion of the PtdInsP_2 _in mixed phosphatidylcholine/phosphatidylethanolamine liposomes [26] and stimulation of the phosphoinositide 5-kinase by an addition of the MgATP [27] greatly potentiates the PLD activation. Direct evidence that phosphoinositide kinase is involved in the PLD activation comes with the use of an inhibitory antibody for this enzyme [26]. The results strongly demonstrate that the PLD activation requires the enhanced PtdInsP_2 _synthesis and the resting cellular levels of PtdInsP_2 _are insufficient for enzyme stimulation. The role of PKC has been determined in the regulation of PtdInsP_2_synthesis in the liver [28] and other cell types [29]. The PKC translocation to membranes and enzyme activation are initial steps in the PLD [for review see [25]] and phosphoinositide 5-kinase [29] stimulation. The lack of PKC/PLD response [24] correlates with the suppressed ability of the L-T_4 _to stimulate the PtdInsP_2 _synthesis in the liver cells of the 720-day-old rats (Fig. 1B). However, in the liver cells of adult animals the L-T_4 _-induced PKC activation and elevation of PtdInsP_2 _synthesis might lead to the PLD activation.

To determine whether the altered thyroid functional status influences the phosphoinositides synthesis, we studied the *myo*-[^3^H]inositol incorporation into the PtdIns, PtdInsP and PtdInsP_2 _in the liver of the drug- and L-T_4 _-treated animals. The MMI administration to the rats was found to be accompanied by the decreased *myo*-[^3^H]inositol incorporation into the PtdIns, PtdInsP and PtdInsP_2 _in the liver (Figure 2A,2B) and drop of thyroid hormones levels in the blood serum (Table 1). The data obtained are consistent with the observations [30] of the drug-induced thyroid gland malfunction. The L-T_4 _injection to the hypothyroid rats increased the T_4 _and T_3 _contents in blood serum (Table 1), the *myo*-[^3^H]inositol incorporation into the PtdInsP_2 _(Figure 2B,2C) and did not changed the levels of the other newly synthesized phosphoinositides in the hypothyroid liver.

Thyroid hormones stimulate lipogenesis in the liver by inducing the enzymes in the lipogenic pathway. The acyl-CoA-glycerol-3-phosphate acyltransferase, which is known to catalyze a rate-limiting step for the synthesis of phosphatidic acid in the rat liver, is dependent on the thyroid gland function [31]. The L-T_4 _administration to the euthyroid rats increased the incorporation of the [^14^C]palmitic acid into the phosphatidic acid and PtdInsP_2 _in the isolated hepatocytes [28]. The results obtained in the present work demonstrated that the L-T_4 _addition to the culture medium significantly increased the mass of phosphatidic acid and did not change the content of PtdIns in the hepatocytes (Table 2). The hormone addition to the culture medium caused the prominent and rapid (within 60 min of cells incubation) increase in the phosphatidic acid synthesis de novo and [^14^C]palmitic acid incorporation into the PtdInsP_2 _and did not change the PtdIns labeling in the isolated hepatocytes [28]. It can be said that the L-T_4 _stimulates the PtdIns precursor synthesis and accumulation in the liver cells in the both long- and short-term manner. The thyroid hormone activation of phosphoinositide synthesis in the liver cells can be supposed to go through an enhancement of the glycerol-3-phosphate acylation, phosphatidic acid accumulation and its conversion into phosphoinositides. Besides, the phosphatidic acid can activate the phosphoinositide 5-kinase [32] and thus stimulate the PtdInsP_2 _synthesis in the hormone-treated liver cells. Considering that the hormone could rapidly (within 60 min) stimulate the phosphatidic acid and PtdInsP_2 _accumulation and did not change the PtdIns content in the liver cells, it could be assumed that in such case the L-T_4 _increases PtdInsP_2 _synthesis via phosphoinositide 5-kinase activation rather than the lipid synthesis de novo.

**Table 2:** Rapid effect of L-T_4 _on phosphatidic acid and phosphatidylinositol contents in the isolated hepatocytes.

**Lipid**	Cells:	
	Control	L-T_4 _-treated
Phosphatidic acid	9,29 ± 0,71	17,7 ± 2,94 *
Phosphatidylinositol	13,6 ± 0,62	13,7 ± 1,83

Hepatocytes were isolated from the liver of adult 90-day-old rats and lipids contents were analyzed as described in "Materials and Methods" and expressed as % of total phospholipids. Hepatocytes were treated with 100 nM NaOH (control) or 10 nM of L-T_4 _for 30 min. Results are mean ± S.E. of six experiments. * p < 0,05 vs. control.

It is well documented that the efficiency of polyphoshoinositide derived second messanger DAG in signaling pathways is closely dependent on the degree of its unsaturation. The unsaturated fatty acids are incorporated into the sn-2 position of the phospholipids by the deacylation-reacylation reactions. Some investigations demonstrated the changes of the fatty acid composition of membraneous lipids at the hypo- and hyperthyroid state of the rats [for review see [33]]. There were generally a reciprocal changes in membranes arachidonic acid contents, namely, a decrease in the hypothyroidism and its increase after the thyroid hormone injection. The L-T_3 _administration to the euthyroid rats increases the saturated fatty acids and arachidonate/linoleate ratio of PtdIns in the liver cell mitochondria [34].

The hypothyroidism was associated with the decrease in the [^3^H]arachidonic acid incorporation into the liver total phospholipids (Figure 3A). The [^14^C]oleic acid labeling of the liver phospholipids was not dependent on the thyroid status of the rats (Figure 3C). The L-T_4_injection to the hypothyroid animals completely abolished the drug-induced changes of the [^3^H]arachidonic acid incorporation into the liver total phospholipids (Figure 3A). The hypothyroidism was associated with the increased arachidonic acid conversion into the prostaglandine E_2 _in the adult rat liver [35]. The L-T_4 _administration to the thyroidectomysed animals reduced the prostaglandine E_2 _synthesis and its content in the liver. Conceivably, the thyroid hormones regulating the arachidonic acid metabolism could maintain the initial level of polyunsaturated phospholipids in the liver cells.

The MMI did not change markedly the ^14^C/^3^H-labeling of PtdIns and PtdInsP_2 _in the liver slices (Figure 3B,3D). The L-T_4 _administration to the rats increased the incorporation of [^3^H]arachidonic acid into the PtdIns and PtdInsP_2 _in the hypothyroid liver (Table 3B) and decreased the content of the oleate-labeled PtdIns in the liver slices (Table 3D). The incorporation of the [^14^C]oleic acid into the PtdInsP_2 _did not differ between the liver slices of control, drug- and hormone-treated animals. As can be seen from the Table 1, the MMI reduced the T_4 _and T_3 _levels in the blood serum, but did not remove completely the hormones from organism. The T_3 _content in the serum of the drug-treated animals was relatively high as compared with control animals. However, the MMI reduced the T_4 _level by 77%. The T_4 _administration to the MMI-treated rats increased significantly T_3 _and T_4 _contents in the serum. It seems possible that the ^14^C/^3^H fatty acids incorporation into the phosphoinositides was rather regulated by the T_3 _than T_4_, although the both hormones are participated in the regulation of the other phospholipids acylation in the rat liver.

## Conclusions

**Figure 4:**
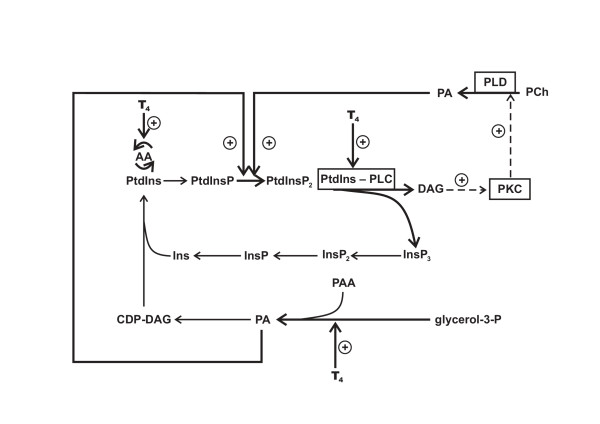
Long- and short-term effects of thyroxine on phosphoinositides metabolism in rat liver. Hormone rapidly and non-genomicaly stimulates PtdInsP_2 _degradation, DAG accumulation and PKC/PLD activation. Hormone-induced PLC activation is followed by PtdInsP_2 _resynthesis, probably via phosphoinositide 5-kinase induction by newly synthesized- or PCh-derived PA. PA could further be converted to polyphosphoinositide precursor – arachidonate-contaning PtdIns under the long lag period of hormone action on the organism. PA – phosphatidic acid, CDP-DAG – cytidine diphosphate diacylglycerol, PAA – palmitic acid, AA – arachidonic acid, Ins – inositol, InsP – inositol 1-monophosphate, InsP_2 _– inositol 1,4-bisphosphate, InsP_3 _– inositol 1,4,5-trisphosphate, PCh – phosphatidylcholine.

The present data have demonstrated that the phosphoinositides synthesis de novo and arachidonic acid incorporation into phospholipids are strongly dependent on the thyroid status of organism. The marked enrichment of animal cell phosphoinositides in arachidonate and the results obtained suggest an important role of the thyroid hormones in the regulation of polyunsaturated PtdIns and PtdInsP_2 _synthesis, which are the predominant substrates of PLC in the numerous signaling pathways. The both long-term and short-term effects of hormone on lipid synthesis have been determined (Figure 4). The L-T_4 _stimulates rapidly and nongenomicaly the PtdInsP_2 _degradation and resynthesis in the liver of the adult rats and does not act on the phosphoinositides metabolism in the cells with the PLC/PKC signaling disability. Besides, the L-T_4 _stimulates the polyphosphoinositides synthesis de novo via the long lag period of time essential to hormone interaction with nuclear receptors and stimulation of protein synthesis [36,37]. Considering that the PtdInsP_2 _participates in the activation of different enzymes (the PLD, protein- and lipid-kinases) involved in the signal transduction in the stimulated cells, the abnormalities of the phosphoinositides synthesis at different pathological states of thyroid gland could disturb other hormones signaling in the target cells.

## Materials and Methods

### Materials

*myo*-[^3^H]-inositol (58 mCi/mmol), [^14^C]oleic acid (58 mCi/mmol) and [^3^H]arachidonic acid (60 Ci/mmol) – Amersham Corp. and [^14^C]CH_3_COONa (25 mCi/mmol) – BPO Isotop (Russia); silica gel from Woelm (Germany). Phosphatidylinositol, phosphatidylinositol 4-phosphate and phosphatidylinositol 4,5-bisphosphate lipid standards were obtained from Sigma (USA). T_4 _and 1-methyl, 2-mercaptoimidazol were from Zdorov'e Trudyaschikhsya (Kharkov, Ukraine). T_4 _and T_3 _radioimmunoassay kits were from Minsk (Belarussia). Other chemicals used were of chemically pure grade.

### Animals

90- and 720-day-old male Wistar rats, which had a free access to food and water and were kept at 24°C on a cycle of 12 h light/12 h darkness were used for experiments. The MMI was injected intraperitoneally (1 mg/100 g weight) in 0.9% NaCl to the experimental animals every day during 16 days-experiment. In some cases, the MMI-treated rats were injected intraperitoneally by T_4 _(200 μg/100 g weight) 48 h prior to killing. Besides, T_4 _(200 μg/100 g weight) was injected to the normal rats 15 and 60 min prior to killing. Control rats received 0.9% NaCl of the same volume. The animals were starved overnight prior to experiment. The thyroid state of rats was monitored by radioimmunological determination of the T_4 _and T_3 _in blood serum.

### Experiments with Liver Homogenates and Slices

The 1 mCi of [^14^C]CH_3_COONa was intraperitoneally injected to rats four times every 30 minutes for 2 hours. The liver was perfused with 0.9% NaCl, then removed and washed in Krebs-Henseleit buffer, pH 7.4, containing 2 mM CaCl_2 _and 0.2% BSA. The pre-labeled liver was used to obtain 10% homogenates and to analyze the^14^C-phosphoinositides. Besides, the slices of unlabeled liver were labeled by incubation in the Eagle medium containing 10% fetal calf serum, 100 units/l streptomycin, 100 units/l penicillin, 13 mg/ml gentamycin and 0.1 μCi/ml of *mio- *[^3^H]inositol or 2.5 μCi/ml of [^14^C]oleic acid or 2.5 μCi/ml [^3^H]arachidonic acid for 1–2 h in 95% O_2_/5% CO_2 _atmosphere at 37°C. The lipids were extracted and analyzed as described below.

### Cell Suspension Experiments

The hepatocytes were isolated from the liver by the method described in [38]. Preparation of hepatocytes was started between 9:00 and 10:00 a.m. The cells (10^7^/ml) were incubated in the Eagle medium containing 10% fetal calf serum, 100 units/liter streptomycin, 100 units/liter penicillin, 13 mg/ml gentamycin and in the presence of 100 nM NaOH (control) or L-T_4_(10 nM) for 30 min in 95% O_2_/5% CO_2 _atmosphere at 37°C. Before lipid extraction, the cells were washed twice with a Krebs-Henseleit buffer pH 7.4, containing 2 mM CaCl_2_, 25 mM HEPES, 0.1% BSA. The lipids were extracted and analyzed as described below.

### Extraction and Separation of Lipids

The phospholipids were extracted according to Folch et al. [39], the phosphoinositides as described in [40]. The chloroform phase was collected and dried under N_2 _at 37°C. The lipids were redissolved in chloroform/methanol (1:2, v/v) and applied on TLC plates. For a total phospholipids isolation, the solvent system: hexane/diethyl ether/acetic acid (80:20:2, v/v) was used, for PtdIns, PtdInsP and PtdInsP_2 _– chloroform/methanol/NH_4_OH (50:40:10, v/v). For phosphatidic acid separation the two-dimensional TLC was used. The TLC plates were developed in chloroform/methanol/ NH_4_OH (60:35:5, v/v) (first direction) and after – in the second direction in chloroform/methanol/acetic acid/water (80:60:7.4:1.2, v/v). The phospholipids masses were determined as described in [41]. The gel spots containing [^14^C/^3^H]lipids were scraped and transferred to the scintillation vials. Radioactivity was measured by a scintillation counter.

## Authors' contributions

NAB conceived of the study and participated in its design, coordination, and manuscript preparation. OAK participated in data collection and performed the statistical analysis.
